# Morphogenesis of sound creates acoustic rainbows

**DOI:** 10.1126/sciadv.ads7497

**Published:** 2025-06-11

**Authors:** Rasmus E. Christiansen, Efren Fernandez-Grande, Ole Sigmund

**Affiliations:** ^1^ DTU Construct, Technical University of Denmark, 2800 Kongens Lyngby, Denmark.; ^2^ DTU Electro, Technical University of Denmark, 2800 Kongens Lyngby, Denmark.; ^3^ DIAC, Universidad Politécnica de Madrid, 28031 Madrid, Spain.

## Abstract

Sound is a crucial sensing element for many organisms in nature, with various species evolving organic structures that produce complex acoustic scattering and dispersion phenomena to emit and perceive sound clearly. To date, designing artificial scattering structures that match the performance of these organic structures has proven challenging. Typically, sound manipulation relies on active transduction in fluid media rather than passive scattering principles, as often observed in nature. In this work, we use computational morphogenesis to create complex, energy-efficient, wavelength-sized single-material scattering structures that passively decompose radiated sound into its spatio-spectral components. Specifically, we design an acoustic rainbow structure with “above unity” efficiency and an acoustic wavelength splitter. Our work demonstrates what is possible when using computational morphogenesis to tailor the emission and reception of sound fields, with relevance to disciplines concerned with the sensing and emission of wave fields.

## Teaser

An acoustic rainbow is realized using computational morphogenesis, enabling energy-efficient spatio-spectral decomposition sound.

## INTRODUCTION

Rainbows, the spatial decomposition of white light into its spectral components, are frequently observed when light propagates through dispersive media—prisms, droplets, or similar (*1*). The analogous phenomenon of spatio-spectral decomposition of sound in free space, where waves oscillating at different frequencies propagate in different directions, is less well known. The decomposition of sound into spectral components can occur in confined media (*2*, *3*), where arrays of resonant structures “trap” sound at different positions in space depending on frequency (*4*, *5*), as in waveguides or reactive silencers (*6*), in solid and/or fluid mixtures (*7*), or in acoustic circulators using nonreciprocity (*8*). Acoustic metamaterials have shown potential for manipulating sound fields (*9*–*11*), using arrays of acoustical (*12*, *13*) or acousto-mechanical resonant structures (*14*) as also exploited by the so-called leaky wave antennas (*15*) or using topological structures (*16*, *17*), which lead to unusual propagation phenomena, including strong acoustic dispersion. Until now, most of these efforts have focused on achieving enhanced directivity at single resonant frequencies or otherwise fairly narrow frequency bands. The controlled spatio-spectral emission of sound, where continuously varying acoustic frequencies are emitted in different directions, has not yet been realized. This is intrinsically a broadband problem, and one where the structural complexity is nearly intractable, as any designed structure operates at the mesoscopic level, potentially having geometric features an order of magnitude smaller than the operating wavelengths as well as features larger (longer) than the operating wavelengths.

Nonetheless, the occurrence of spatio-spectral decomposition of sound (not requiring unusual acoustic dispersion or exotic propagation phenomena) can be observed in nature, where living organisms use sound to relate to their environment. Examples include the mammalian pinnae (*18*, *19*), noise emitting bats (*20*), cetacea (*21*–*23*), and others (*24*). The outer ear of primates is a close example to our species (*25*): Its intricate geometry produces complex interference phenomena, which result in unique spatio-spectral cues that aid the localization of sound sources. Some man-made structures have to some extent successfully reproduced natural ones, mimicking the natural design—such as whales and purpoises (*26*, *27*). However, to date, man-made structures have failed to achieve a tailored and controllable spatio-spectral decomposition of sound, based on scattering principles, as commonly found in nature. This knowledge gap motivates our current efforts.

**Fig. 1.**
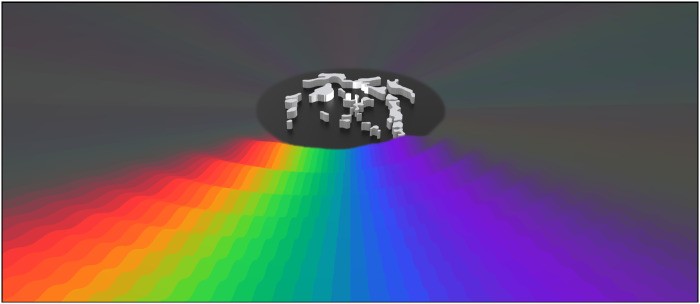
Visualization of the ARE. The morphogenetical topology optimization method shapes the scattering inclusions, shown as gray material. When the ARE is excited by monopolar source emitting broad-band white noise, the radiated sound creates an acoustic rainbow. The source is positioned at the center of the emitter (illustrated using white light) and driven with equal power at all frequencies from 7600 to 12800 Hz. In the figure, the experimentally measured acoustic output (far field) is mapped to the visible spectrum of light by its magnitude and frequency content in the full 360° surrounding the ARE (see the Supplementary Materials for a detailed description).

**Fig. 2.**
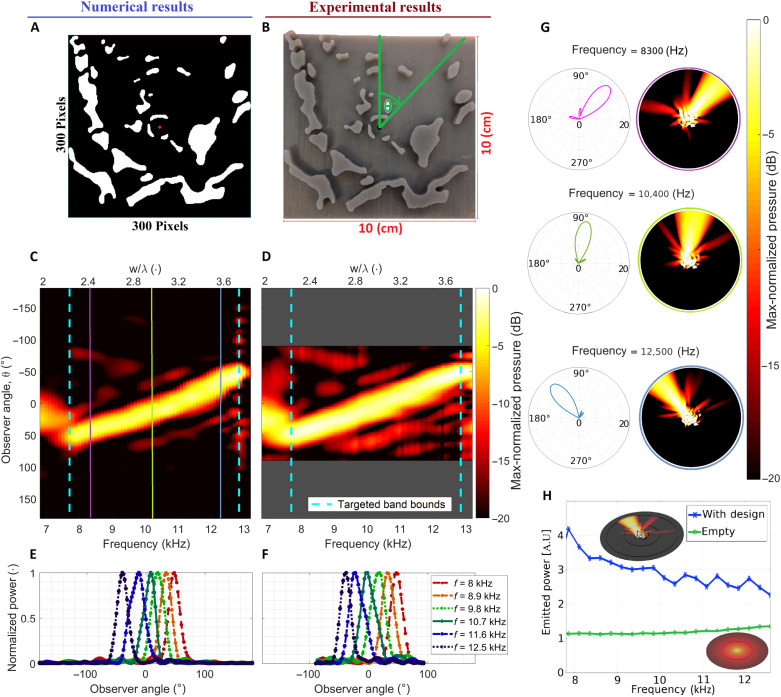
ARE geometry and emission profile with the left (middle) column showing numerical (experimental) results. (**A**) Device blueprint with the monopolar source position indicated by the red dot. (**B**) Experimental realization of the ARE (3D printed), the sample size, and angular convention are overlaid. (**C** and **D**) Maps of the angle-dependent max-normalized far-field sound pressure with (C) simulated and (D) experimental results showing remarkable agreement (see the Supplementary Materials for the data treatment procedure). (**E** and **F**) Per frequency max-normalized far field power. (**G**) Simulated far- (left) and near-field (right) sound pressure levels for three frequencies indicated by the vertical lines in (C) on a 20-dB scale. (**H**) Total emitted power (in arbitrary units) from the monopolar source when placed in the ARE (blue curve), relative to its free-space emission (green curve), revealing above unity efficiency.

**Fig. 3.**
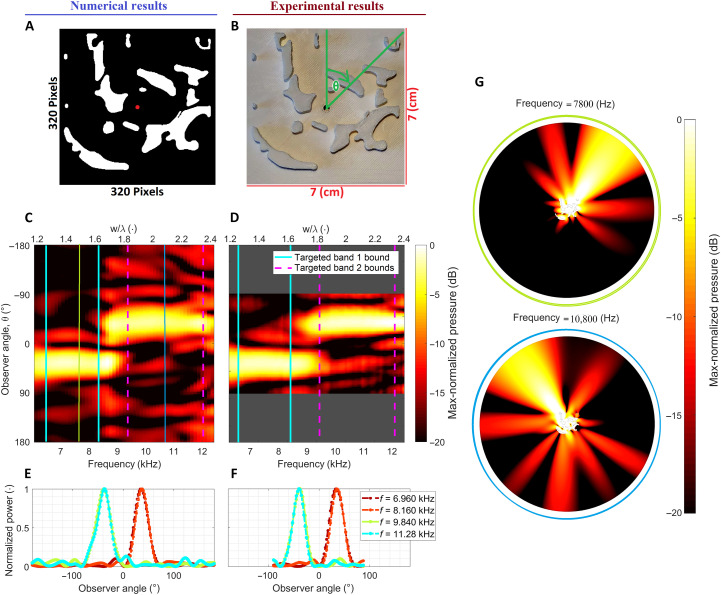
Lambda splitter geometry emission profile with the left (middle) column showing numerical (experimental) results. (**A**) Blueprint for the lambda splitter with the red dot indicating the source position. (**B**) Experimentally constructed sample (3D printed) with corresponding dimensions. (**C** and **D**) Max-normalized far-field sound pressure maps. The cyan and magenta vertical lines indicate the bounds of the frequency bands targeted in the design process. Most of the energy of all spectral components are observed to be directed into the +35° direction or the −35° direction depending on frequency. (**E** and **F**) Per frequency max-normalized far-field sound power, showing a clear decomposition of the spectral components. (**G**) Simulated near-field sound pressure for two frequencies indicated by the vertical lines in (C).

Our work focuses on a general unifying framework that enables the design of structures that fully control the spatio-spectral decomposition of emitted/received sound as illustrated in Fig. 1. We present a single-material passive acoustic scattering structure, systematically tailored using computational morphogenesis to convert a broadband white-noise signal emitted by a single monopolar source into an acoustic rainbow. Note that power is radiated more efficiently from the source in the device across the entire targeted frequency band than if the source would emit into free space. That is, we have tailored a device which simultaneously shapes the emission pattern and maximizes the emission efficiency (Fig. 2). To highlight the versatility of the method, we use the same approach to design a lambda splitter (Fig. 3) capable of dividing a broadband signal into two well-defined spatio-spectrally separated frequency bands. Both structures are based solely on the scattering of sound from hard surfaces and work across 50% frequency bands under free field conditions. Through experiments, we show close agreement between the numerical predictions and real world operation, demonstrating the strength and accuracy of the design method. The Supplementary Materials includes videos of simulations illustrating the workings of the two devices, showing the near-field emission patterns as a function of frequency and illustrating the acoustic rainbow as heard by an observer at different observation angles.

Because of the reciprocity of the physical system, the properties of emission and reception may be trivially reversed. Our work thus demonstrates the power of using computational morphogenesis to tailor the emission and reception of sound fields. The results presented in the following are also of relevance to other disciplines concerned with the sensing and emission of wave fields.

Our work takes advantage of the rapid growth in computational power, which in recent years has enabled large-scale modeling and synthesis of sound as a full-wave phenomenon in complex domains and geometries. Further, advances in modeling and production techniques have enabled the use of morphogenetic methods such as topology optimization (*28*) in the design of practically realizable structures, ranging in size from decameters to nanometers (*29*–*33*). Topology optimization has previously been applied in acoustics to tailor phononic crystals and metamaterials (*34*), designing for effective negative material parameters (*35*, *36*), tailoring hyperbolic metamaterials (*37*) and topological insulators (*38*, *39*), as well as for acoustic-structure interaction problems (*40*) for filtering (*41*) and sound insulation (*42*) among others. Topology optimization, combined with accurate modeling of the wave nature of sound (*43*) and the availability of advanced production techniques like additive manufacturing, provides nearly unlimited design freedom, enabling the design of meta-materials and nonintuitive structures hitherto deemed realizable.

Inspired by the natural occurrences of passive “sound-shaping” devices, we modify our earlier morphogenetic design framework (*44*) for tailoring passive acoustic scattering structures with dimensions on the order of a few wavelengths, to also control the near- and far-field radiation pattern depending on the frequency content of the sound field. The modified framework is set up to design scattering structures, which exert precise spatio-spectral control of the radiation and simultaneously improve the impedance matching between the source and surrounding medium, acting as an acoustic antenna, resulting in greater acoustic power output than the free-space emission of the monopolar source (see Fig. 2H).

In short, our design approach consists of an iterative process of redistributing acoustically reflecting material in an air background inside a specified region of space $\Omega_{D}$ to match the sound field emitted from the device under design ∣p(x,ω)∣ to a desired target emission pattern $|\;p_{\text{target}}\left(x,\omega \right)\;|$ elsewhere in space $\Omega_{t}$ , across a specified frequency band $\omega \in \left[\omega_{1},\omega_{2}\right]$ . A measure of how well this goal is achieved may be written as the figure of merit (FOM)

$$\Phi \left(\omega \right)=\int_{\Omega_{t}}{\left[{|p\left(x,\omega \right)|}^{2}-{|p_{\text{target}}\left(x,\omega \right)|}^{2}\right]}^{2}\;dx$$ (1)

Assuming continuous pressure fields, it is apparent that Φ(ω)=0 if and only if $|\;p\left(x,\omega \right)|\;=|\;p_{\text{target}}\left(x,\omega \right)\;|$ for all $x\in \Omega_{t}$ . Thus, minimizing Φ(ω) must result in ∣p(x,ω)∣ approaching $|\;p_{\text{target}}\left(x,\omega \right)\;|$ . Using this FOM, we use adjoint sensitivity analysis to obtain gradient information on how the FOM changes with changing device geometry (*45*). We then recast the design problem as a constrained optimization problem with the goal of minimizing Φ(ω) over the frequency range $\omega \in \left[\omega_{1},\omega_{2}\right]$ (*28*) using a minimax formulation. To solve the optimization problem, we use an iterative design update scheme exploiting the gradient-based optimization algorithm, the method of moving asymptotes (*46*) to efficiently tailor a device to emit sound in one of a wide range of unusual patterns and magnitudes determined by the selection of $p_{\text{target}}\left(x,\omega \right)$.

To accurately model the sound pressure as a function of frequency and material layout, we use the following model for the physics. We assume time-harmonic field behavior in the finite domain $\Omega \in {\mathbb{R}}^{n}$ ( n∈{1,2,3} ), containing both $\Omega_{D}$ and $\Omega_{t}$ as subsets. In the two cases studied in the following, we perform the modeling and optimization based on a pure two-dimensional (2D) model (i.e., $\Omega \in {\mathbb{R}}^{2}$ ) while also validating the results using full 3D analysis. The numerical modeling is carried out using the inhomogeneous Helmholtz Eq. 2

$$\begin{array}{c} \nabla \cdot \left[\frac{1}{\rho \left(x\right)}\nabla p\left(x\right)\right]+\frac{\omega^{2}}{\kappa \left(x\right)}p\left(x\right)=i\omega \rho Q\delta \left(x-x_{0}\right)\;\forall x\in \Omega \end{array}$$ (2)

Here, ρ(x) denotes the density, κ(x) the bulk modulus, ω the angular frequency, *p* the pressure, and x the spatial position in Cartesian coordinates. ∇ denotes the spatial gradient operator and · the dot product, *Q* denotes the volume velocity and $\delta \left(x-x_{0}\right)$ the Dirac delta function, with the right-hand side of the equation corresponding to a monopolar source placed at $x_{0}$ (*43*). The model domain Ω is truncated along its boundary δΩ using an approximation of the free field Sommerfeld radiation condition

$$\begin{array}{c} \underset{|r|\to \infty }{\text{lim}}\left\{|\;r\;|\cdot \left[\frac{\partial p\left(r\right)}{\partial r}+i\frac{\omega }{c\left(\rho ,\kappa \right)}p\left(r\right)\right]\right\}=0 \end{array}$$ (3)

Here, $\frac{\partial }{\partial r}$ denotes the partial derivative, i the imaginary unit, $\underset{|r|\to \infty }{\text{lim}}$ the limit operation, *r* the spatial position in Polar coordinates, and *c* the speed of sound.

We assume that any mechanical vibrations in the device are negligible, as the structure is rigid, and thus no mechanical modeling of the solid material is used. Instead, the solid is modeled as an extremely dense liquid ( $\rho_{\text{solid}}\gg \rho_{\text{air}}$ ) with extreme bulk modulus ( $\kappa_{\text{solid}}\gg \kappa_{\text{air}}$ ) as compared to the air background. The validity of this simplified approach under the given assumptions on the material parameters has previously been experimentally verified (*47*).

In this work, we consider a 2D spatial setting (*n* = 2) for simplicity; however, given sufficient computational resources, neither the numerical design approach nor the practical realization of the designed devices hinders an extension to three dimensions. Noting here that 3D sound fields are more complex in nature compared to 2D sound fields. The prescribed maximum size of the design dictates the minimum frequency at which it can effectively operate when relying on scattering. We considered structures with a size of 10 cm by 10 cm and smaller (similar to the mammalian pinnae), which can operate effectively from ≈4 kHz and upward. Given dispersion free materials, any designed structure can be isotropically rescaled to shift the operational bandwidth. A detailed description of the design procedure, optimization problem, numerical methods used for simulations, the experimental setup, and the measurement procedure, along with animations and audio examples, is provided in the Supplementary Materials and references therein.

## RESULTS

### The acoustic rainbow emitter

Figure 1 presents the experimentally measured sound power emitted from a single centrally placed source and shaped by the acoustic rainbow emitter (ARE) designed using the approach detailed in the Supplementary Materials by minimizing the FOM in Eq. 1 for 20 equidistant frequencies across the interval of 7.6 to 12.8 kHz. The illustration of the measured sound power is made by mapping the sound field according to its angle-dependent frequency content to the visual spectrum of light, superimposed on waves with appropriately scaled wavelengths.

**Fig. 4.**
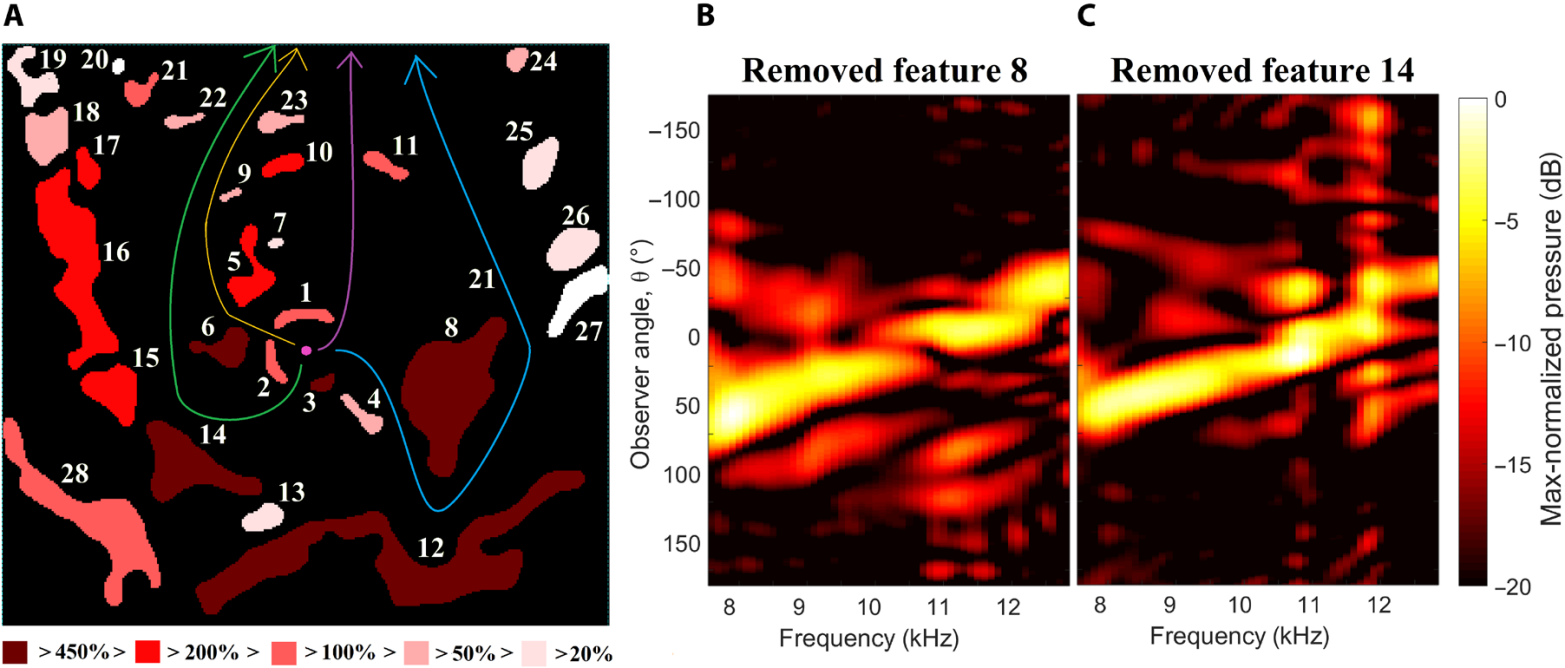
Sensitivity study, revealing the effect on Φ of removing each of the 28 ARE fatures. (**A**) ARE blueprint with numbering of the 28 features and a colormap illustrating the deterioration of Φ as each single feature is removed showing that (almost) all features have importance for Φ . The green, orange, purple, and blue colored lines sketch four propagation paths from the source (pink) to the front (θ = 0) of the device. (**B** and **C**) Far-field sound pressure map for the ARE with (B) feature 8 and (C) feature 14 removed, respectively. These maps should be compared to the original ARE emission pattern in Fig. 2C.

The device blueprint for the ARE is presented in Fig. 2A with solid (void) regions shown using white (black) and the monopolar source position indicated by the red dot. The blueprint consists of numerous intricately shaped sound-hard features among which are found three centrally placed reflectors near the source, guiding its emission through three openings of different widths. A reflector-like ridge with six narrow channels through it is situated behind and to the left of the source. This ridge serves to reflect and guide the sound waves in the specified directions, as the channels create acoustic paths of different lengths, which dictate how the waves interfere depending on their frequency to progressively guide waves of lower frequency toward the right (+θ) and of higher frequency toward the left (−θ). Because of the complex interplay between the many design features, it is extremely difficult (if not functionally impossible) to fully isolate the effect of any individual feature. It is however clear that the individual features are working cohesively, across different spatial scales and thus being able to act on the sound field in a broadband sense, enabling the spatio-spectral modification of sound emission. It is later shown, as hypothesized, that removing any one individual feature changes the radiation pattern (Fig. 4). Despite the above, it is nonetheless possible to identify physical principles of operation, as is presented and analyzed in the next section. Figure 2B presents an image of the 3D-printed experimental specimen with the physical dimensions and the adopted angular convention overlaid. Figure 2C presents the numerically simulated radiated sound pressure magnitude clearly revealing the rainbow emission pattern, with the angle of maximum emitted acoustic power varying continuously from −50° to 50° as the frequency shifts from 7.6 to 12.8 kHz. The vertical cyan dashed lines specify the bounds of the frequency band targeted in the design process. From the data, it is clear that the spatial separation of the spectral components is successful, achieving differences in acoustic power of at least one order of magnitude between the main emission lobe and any side lobes. Comparing the simulated data to the experimental measurements shown in Fig. 2D, clear agreement is observed. In both the simulated and measured data, weak side lobes are observed. These are caused by a fraction of the emitted acoustic energy not being effectively scattered in the prescribed direction by the optimized design. In addition to the simulation data presented in Fig. 2C, we evaluated the ARE numerically using a 3D model of an extruded and sandwiched version of the device, including thermoviscous boundary layers on all surfaces to model boundary losses. Using this model, we observe a relatively small (near) angular independent loss. The loss has a frequency-dispersive magnitude ranging from 1 dB at 7000 Hz to 2 dB at 13000 Hz. Note that as the ARE does not rely on resonant phenomena to operate, this loss has (nearly) no effect on the emission pattern, which, in the lossy model, is found to agree with the pattern in Fig. 2C. The normalized far-field power, computed using the Helmholtz-Kirchhoff integral method, as a function of emission angle, for six equidistant frequencies, is presented in Fig. 2E (simulation) and Fig. 2F (experiment), respectively. The regular angular shift in the emission direction as a function of frequency is clearly observed, as is a substantial difference in main to side lobe power of approximately 20 dB. To further illustrate spatio-spectral decomposition of the sound, three representative near-field pressure maps at 8300 Hz (purple line in Fig. 2C), 10,400 Hz (green line in Fig. 2C), and 12,500 Hz (blue line in Fig. 2C) are plotted in Fig. 2G showing how the emission pattern changes with frequency along with polar plots of the far-field pressure distribution, clearly showing that both the near- and far-field exhibit the desired spatio-spectral response. Note that there is nothing that hinders selecting a function of the far-field as the FOM or selecting a function of both the near- and far-field for that matter. In addition to achieving the desired spatio-spectral response, the ARE achieves “above unity” efficiency across the entire frequency band of operation in terms of the total sound power emitted by the source relative to the source radiating into free space, as shown in Fig. 2H. This plot also reveals the nonresonant response of the ARE (see also fig. S2 and the associated text in the Supplementary Materials for details). The high efficiency is obtained by the morphological design method as it implicitly matches the impedance of the source to the surrounding medium through the scattering structures constituting the ARE to match the prescribed target field. Such above unity efficiency may be contrasted with previous work on the design of an acoustic prism using an array of acousto-mechanical resonant structures (*14*), where an efficiency of a few percent was reported. Our results thus prove that the proposed design framework enables the realization of highly efficient spatio-spectral decomposition of sound through passive acoustic scattering from systematically engineered single-material acoustically hard surfaces, as the emission/reception properties are tailored through a target FOM.

### Feature sensitivity and physical principle of operation

The complex geometries of the optimized devices, coupled with the wave nature of the physics, mean that providing a simple interpretation of the effect of any individual feature on the device performance is functionally impossible. One could be tempted to claim that the designs features are simply randomly distributed and question their importance. This is however not the case.

To demonstrate this claim, we evaluate the importance of each of the 28 subwavelength features constituting the ARE, by systematically removing the features one-by-one and compute the resulting deterioration of Φ . The results of this investigation are reported in Fig. 3A reporting the relative deterioration (i.e., increase) of Φ as each feature is removed. The effect of removing the features in turn is seen to range from deteriorating Φ by a few percent (e.g., removing feature 27) to a deterioration by more than 450% (e.g., removing feature 6). For reference, the removal of the entire ARE (corresponding to having the point source emit into free space) results in a ≈325% increase of Φ , while removing the source (corresponding to turning off the device) results in ≈350% deterioration. That is, remarkably Φ is more sensitive to the interplay between some of the design features compared to removing the ARE entirely or turning off the sound, revealing the importance of the interplay between the components constituting the device. From Fig. 4A, it is seen that generally the most important features are the largest, the ones situated closest to the source, and the features that form the anterior of the ARE. Examples of the effect of removing individual features on the emission pattern are presented in Fig. 4B (feature 8) and Fig. 4C (feature 14). Comparing these pressure maps to the one for the optimized device in Fig. 2C, it is clearly seen that the changes to the radiation pattern are quite complex. Removing feature 8 affects the emission pattern across most of the frequency band of operation, particularly raising the sound pressure around the 100° emission angle, the angle at which feature 8 is situated. Meanwhile, removing feature 14 is seen to mainly affect the pattern at frequencies above 10 kHz.

The features at the anterior of the ARE in-part serve to reflect the sound emitted by the source, hereby reducing transmission behind the ARE, as seen from the frequency-independent ≈20 dB drop in sound pressure in the angular range below −90° and above 90°. In addition, these features cause a frequency-dependent change to the acoustic propagation path length (i.e., the distance that a sound wave travels around the features) and an associated phase shift of the sound wave (or delay) induced by the propagation path. The smaller features close to the source cover a larger part of the solid angle as seen from the source relative to the other features, hereby through scattering modifying the phase and path length of a fraction of the field emitted from the monopolar source, before the sound propagates further through the device.

Removing the smaller features situated above and furthest away from the source are generally seen to have the smallest deteriorating effect on Φ . Their small size and large distance from the source mean they effectively have a smaller angular size as seen from the source point than their larger counterparts. In turn, this means that they are exposed a smaller fraction of total acoustic power emitted from the source, compared to the larger features. Thus, they will have a comparatively smaller effect on the total sound propagation.

On the basis of our analysis, we conclude that the physical operation principle exploited by the design is the spectrally dependent phased interference of sound waves that are scattered as they travel around the different elements/features of the design. Thus, the device does not operate based on a (series of) resonance(s), as evidenced by the fact that, when exited, the ARE shows no sign of resonant response (see the total emitted acoustic power as a function of frequency in Fig. 2H). The fact that the ARE does not rely on resonances to shape the emission pattern, nor includes long narrow regions, makes the effect of visco-thermal losses at material interfaces less critical than in resonant devices relying on the local build-up of acoustic energy. The losses observed are like those seen in conventional sound scattering from rigid objects.

It is observed that soundwaves are guided along multiple different paths of different lengths, and it is the interference between waves traveling through these different channels (and individual features) that results in the desired spatio-spectral decomposition. Specifically, four main channels (sketched in Fig. 2A) are observed in the design, each of them of different lengths, on the left side of the point source (−40°), behind it (±180°), and two on the right side (both starting at +45°). Sound waves propagating along these paths of different lengths will interfere constructively and destructively, depending on their relative phase, much like the operational principle behind phased active arrays. However, because the present design is entirely passive, relatively intricate propagation paths are necessary. In addition, the specific shape of the features in the design influences the scattering pattern of the sound, which is essential for achieving precise and controlled spatio-spectral decomposition. To grasp the operation of the device in a simplified setting, it is possible to analyze the emission directions at different frequencies. In the frontal emission direction (θ = 0°), it is seen that the travel path difference between waves propagating along one of the paths to the right side of the source and the path to the left side is ~3 cm, which results in a 2π rad difference at 10 kHz. Thus, there is constructive interference between the waves traveling the two paths for the said frequency in this direction. At 8 and 12 kHz, the path difference in the frontal direction results in phase differences of 1.6π rad and 2.5π rad, respectively, resulting in partial cancellation through negative interference. This same analysis is conducted for the other directions of emission, to confirm the basic physical underpinnings of the device. That said, and as discussed in Introduction, the complexity of the problem is very high, as it operates over a broad frequency band and at disparate spatial scales. Therefore, in terms of analyzing the effect of individual features on the performance, we find that the sensitivity analysis performed above provides more information on the influence of removing different features of the design, which also showed that the most sensitive features are those that fundamentally define the main propagation paths discussed above.

### The acoustic lambda splitter

Spatio-spectral decomposition of sound can be tailored as desired using the proposed design approach. To demonstrate this, we also synthesize a free-space broadband acoustic lambda splitter. This device separates the sound emitted by a source in two distinct spatial directions, depending on the spectral content of the waves. The lambda splitter is designed to emit sound in the +35° direction for frequencies in the band from 6.5 to 8.4 kHz and the −35° direction for frequencies in the band from 9.4 to 12 kHz. The resulting blueprint for the lambda splitter and a 3D-printed realization are shown in Fig. 3 (A and B), respectively. Maps of the radiated sound pressures are seen in Fig. 3C (simulation) and Fig. 3D (experiment). The emitted sound is seen to be separated into the two targeted spatial directions according to the frequency content (the frequency band limits are highlighted using cyan solid and magenta dashed lines). Again a substantial main to side lobe difference is observed for the sound pressure inside the targeted bands of operation. Across the low-frequency band, more than 88% of the power emitted over the measured −90° to 90° interval is directed into the main lobe at full width at half maximum. For the high-frequency band, 82% of the total power is emitted in the target direction. In practice, this is a clearly detectable decomposition of the sound field. Figure 3E (simulation) and Fig. 3F (experimental) show the max-normalized far-field power as a function of emission angle for four frequencies, two inside each frequency band. To illustrate the directional emission of the sound, two representative near-field pressure maps at 7800 Hz (green line in Fig. 2C) and 10,800 Hz (blue line in Fig. 2C) are plotted in Fig. 2G, showing how the direction of emission changes with frequency. The emission direction of the main lobe is well separated for frequencies in different bands and overlaps almost perfectly for frequencies in the same band.

Note that the presented designs are non-unique in the sense that several different design topologies are found to lead to very similar responses in terms of minimizing the goal function. This is expected and often observed when applying optimization techniques to solve inverse design problems as they are (almost) all nonconvex in nature.

## DISCUSSION

The devices and method presented in this work are of direct relevance to acoustics and the principles applicable to other fields concerned with wave propagation such as optics and radio backscattering. The design and experimental realization of these devices reveal the potential for manipulating the spatio-spatial properties of sound fields based solely on passive scattering elements. The work demonstrates a powerful approach that could be used to aid in the understanding of the acoustic radiation and scattering processes found in complex natural structures.

When inspecting the geometry and topology of the device, several of its features are reminiscent of features present in the pinnae whose function is known to be the spatio-spectral decomposition of sound. Specifically, features 14 to 19 of the device shown in Fig. 4 resemble the acoustic channel formed by the helix—which is known to be an important element of the lower-frequency range and the first notch in the head-related transfer function (*48*, *49*). Also, the tragus area, proximal to the entrance of the ear canal, is known to be important at higher frequencies (*48*), similarly to features 1 to 3 in Fig. 4, close to the position of the source.

Both sample applications of controlled morphogenesis of sound feature scattering structures that show distinct angular geometric asymmetries. These asymmetries account for the changing length of the sound propagation paths as a function of direction, leading to the interaction phenomena required for the spatio-spectral decomposition of sound in the mesoscopic wavelength range. The utilization of a complex interplay between a set of passive scattering structures resulting in nonresonant spatio-spectral sound manipulation with high efficiency opens an avenue for reducing the energy use now associated with resonance-based and active sound manipulation.

## MATERIALS AND METHODS

The design method is implemented using an in-house MATLAB code, where the physics is modeled using an implementation of the Helmholtz equation (*43*). The frequency responses of the optimized structures are evaluated with COMSOL Multiphysics (*50*) using both 2D and full 3D models. The ARE and lambda splitter were fabricated in hard plastic using 3D printing. Details about the proposed morphological design method, the numerical model, the experimental setup and experimental procedure, the procedure for creating Fig. 1, as well as a set of video and audio examples of the sound emission from the two devices are available in the Supplementary Materials.

## Supplementary Materials

### The PDF file includes:

Supplementary Text Figs. S1 to S3 Legends for movies S1 to S4 References
