# Supplementary material for: Morphogenesis of sound creates acoustic rainbows
Source: Sci Adv. 2025 Jun 11;11(24):eads7497. doi: 10.1126/sciadv.ads7497 (PMC12153974; doi:10.1126/sciadv.ads7497)
Supplement: Supplementary file 1 — Supplementary Text Figs. S1 to S3 Legends for movies S1 to S4 References [file sciadv.ads7497_sm.pdf]

Science Advances
AAAS

Supplementary Materials for

Morphogenesis of sound creates acoustic rainbows

Rasmus E. Christiansen *et al*.

Corresponding author: Rasmus E. Christiansen, raelch@dtu.dk

Sci. Adv. **11**, eads7497 (2025)
DOI: 10.1126/sciadv.ads7497

The PDF file includes:

Supplementary Text
Figs. S1 to S3
Legends for movies S1 to S4
References

Other Supplementary Material for this manuscript includes the following:

Movies S1 to S4

Supplementary Text:

Animations and audio examples. Animations of the near-field pressure around the acoustic rainbow emitter (ARE) and lambda-splitter as a function of frequency, are provided as supplementary movies S1 and S2, respectively. These animations show how the acoustic near-field pressure changes with excitation frequency. From S1 of the ARE the smooth change in the main emission direction from 50° to -50° as the driving frequency is varied from 7600 Hz to 12800 Hz is clearly observed. From S2 the lambda-splitter the abrupt jump from emitting in the 35° direction to the -35° direction is likewise clearly observed.

Two audio-video illustrations, for the ARE driven at four frequencies simultaneously, are provided in the supplementary movies S3 and S4. Note that for these audio illustrations, the excitation frequencies are down-sampled by a factor of 10 to aid perception and ensure that standard loudspeakers are capable of accurately reproducing the audio. The down-sampled frequencies used to drive the rainbow are $f \in \{820\ \mathrm{Hz}, 966\ \mathrm{Hz}, 1113\ \mathrm{Hz}, 1260\ \mathrm{Hz}\}$. Movie S3 shows the rainbow performing a 360° rotation relative to a stationary observer indicated by a red dot. The acoustic far field power is visualized by mapping it to the visible spectrum according to its angular frequency content, following the same procedure as for Fig. 1. As the rainbow rotates, audio is played in accordance with the sound field experienced by the stationary observer. Movie S4 shows the map presented in Fig. 2c with the addition of the four green dashed lines indicating the excitation frequencies. Audio is played in accordance with the audio

heard by an observer at the angular position indicated by the sweeping cyan line. The audio is generated using far-field pressure data obtained from the numerical model for the ARE, discussed next.

Numerical modelling of the final optimized devices. The angular and frequency dependent emission patterns from the ARE and lambda-splitter shown in Fig. 2c and Fig. 3c, have been computed using Helmholtz-equation-based models created in COMSOL Multiphysics *(50)*. Here all structural members of the devices is modeled as sound hard boundaries, perfectly reflecting any impinging sound. A mono-polar point source is used as the sound emitter and the model domain is truncated using perfectly matched layers. COMSOL models along with .mphbin files containing the geometries of the ARE and lambda-splitter are available from the corresponding author upon reasonable request. These models allow any user to directly execute the study denoted “Frequency Sweep Across Operational Band”, after which the near- and far-field pressure and intensity distributions can be studied in predefined plot groups. The software program COMSOL Multiphysics and a license for the acoustic module is required to execute the models.

Model domain. In the design process, the physics is modelled in a rectangular model domain $\boldsymbol{\Omega}$ (fig. S1) with a subdomain constituting the design domain, $\boldsymbol{\Omega}_{\mathbf{D}}$, in which the structure under design is situated and another subdomain constituting the target domain, $\boldsymbol{\Omega}_{\mathbf{D}}$.

Acoustics model. The acoustic problem is modeled in $\boldsymbol{\Omega}$ in the frequency domain using the inhomogeneous Helmholtz equation *(47)*,

$$\nabla \cdot \left(\frac{1}{\rho(\boldsymbol{x})} \nabla p(\mathbf{x})\right) + \omega^2 \frac{1}{\kappa(\boldsymbol{x})} p(\boldsymbol{x}) = \mathrm{i}\omega\rho Q\delta(\boldsymbol{x} - \boldsymbol{x}_0) \ \ \forall \ \boldsymbol{x} \in \boldsymbol{\Omega}. \quad \textbf{(S1)}$$

Here $\rho(\boldsymbol{x})$ denotes the density, $\kappa(\boldsymbol{x})$ the bulk modulus, ω the angular frequency, p the pressure and $\boldsymbol{x}$ the spatial position in Cartesian coordinates. ∇ denotes the spatial gradient operator, $\cdot$ the dot product, Q denotes the volume velocity and $\delta(\boldsymbol{x} - \boldsymbol{x_0})$ Diracs delta function. The domain is truncated along the boundary $\delta\boldsymbol{\Omega}$ and the free field Sommerfeld radiation condition

$$\lim_{|r|\to\infty} \left(|r|^{\frac{1}{2}} \cdot \left(\frac{\partial p(r)}{\partial r} + \mathrm{i}\,\frac{\omega}{c(\rho,\kappa)} p(r)\right)\right) = 0 \quad \textbf{(S2)}$$

is approximated through a boundary condition on $\delta\boldsymbol{\Omega}$. Here $\frac{\partial}{\partial r}$ denotes the partial derivative, i the imaginary unit, $\lim_{|r|\to\infty}$ the limit operation, r the spatial position in Polar coordinates, and c the speed of sound.

No mechanical model of the solid material constituting the devices is used. Instead, the solid is modeled as a highly dense liquid ($\rho_{\mathrm{solid}} \gg \rho_{\mathrm{air}}$) with extreme bulk modulus ($\kappa_{\mathrm{solid}} \gg \kappa_{\mathrm{air}}$), as compared to the air background thus neglecting transverse waves in the solid material; the validity of this simplified approach has previously been experimentally verified *(28,32)*.

A hybrid Finite-Element and Wave-Based (WBM-FEM) method is used to discretize equations **(S1-S2)** for the design procedure *(51)*, resulting in a linear system of equations which is solved to obtain the pressure,

$$\boldsymbol{S}(\rho, \kappa, \omega)\, \boldsymbol{p} = \boldsymbol{f}(\omega) \qquad \textbf{(S3)}$$

where $\boldsymbol{S}$ is the material and frequency dependent system matrix, $\boldsymbol{p}$ is the pressure vector, with components p_l, and $\boldsymbol{f}$ is the acoustic load vector. An approximation of the pressure field is constructed from $\boldsymbol{p}$ and the chosen basis $\psi_l(\boldsymbol{x})$ as,

$$p(\boldsymbol{x}) = \sum_l p_l\, \psi_l(\boldsymbol{x}) \qquad \textbf{(S4)}$$

In brief, the Wave Based Method (WBM) (*52*), uses free-space solutions of the Helmholtz equation in homogenous convex domains as basis-functions on which the pressure field is approximated. This approach leads to very small (dense) linear equation systems that are computationally expensive to set up compared to the equations systems obtained using lower order finite-elements. However, given the small size, the computational complexity associated with the solution of the resulting linear system of equations is significantly lower for the WBM than for the FEM. Therefore, modelling using the WBM is computationally cheaper than using FEM, but requires a convex domain with homogeneous material parameters to work. The hybrid WBM-FEM method consists of combining the WBM model applied to convex-subdomains with homogeneous material parameters and the FEM to the remaining domains (the design domain in our case), and then connecting the different domains using an interface condition exploiting Lagrange multipliers. An added benefit of performing optimization using the hybrid WBM-FEM is that the setup cost of the WBM sub-systems is only done once, as these domains, and their material contents, do not change during the optimization. Therefore, the overhead associated with setting up the WBM

systems is almost negligible when compared to the hundreds of system solutions needed throughout the optimization.

Topology optimization. The problem of designing devices for the spatio-spectral decomposition of sound is formulated as a topology optimization problem. The goal, or objective, of the problem is to determine a spatial material configuration constituting the device, which best approximates the desired emission profile, given a specified excitation. The discrete material distribution problem is transformed into a continuous problem by introducing the mathematical design field, $\xi(\boldsymbol{x}) \in [0,1]$, which is discretized into a piecewise constant field with one design variable $\xi_k \epsilon [0,1]$ assigned to each finite element in the design domain. The discretized design field is then used to smoothly interpolate the density and bulk modulus in each element between air and a reference "solid", using the interpolation functions

$$\rho(\xi(\boldsymbol{x}))^{-1} = {\rho_{\mathrm{air}}}^{-1} + \mathrm{F}\big(\xi(\boldsymbol{x})\big)({\rho_{\mathrm{solid}}}^{-1} - {\rho_{\mathrm{air}}}^{-1}), \qquad \textbf{(S5)}$$

$$\kappa(\xi(\boldsymbol{x}))^{-1} = {\kappa_{\mathrm{air}}}^{-1} + \mathrm{F}\big(\xi(\boldsymbol{x})\big)({\kappa_{\mathrm{solid}}}^{-1} - {\kappa_{\mathrm{air}}}^{-1}). \qquad \textbf{(S6)}$$

Here F denotes a filtering operator applied to the design field in order to drive the material distribution towards a physically admissible binary distribution, gradually during the design process, as detailed in *(28)* and references therein. The parameter values for the density and bulk modulus chosen for the air and the solid are: $\rho_{\mathrm{air}} = 1.204 \frac{\mathrm{kg}}{\mathrm{m}^3}, \kappa_{\mathrm{air}} = 142 \cdot 10^3 \frac{\mathrm{N}}{\mathrm{m}^2}, \rho_{\mathrm{solid}} = 2643 \frac{\mathrm{kg}}{\mathrm{m}^3}, \kappa_{solid} = 6.87 \cdot 10^{10} \frac{\mathrm{N}}{\mathrm{m}^2}$, respectively.

The continuous structural optimization problem is states as:

$$\underset{\xi}{\text{minimize}}\ \underset{\omega_j}{\text{maximum}} \left(\int_{\boldsymbol{\Omega}_\mathrm{t}} \left(\left| p\left(\mathrm{F}\left(\xi(\boldsymbol{x})\right), \omega_j\right) \right|^2 - \left| p_\mathrm{target}\left(\mathrm{F}\left(\xi(\boldsymbol{x})\right), \omega_j\right) \right|^2 \right)^2 \mathrm{d}\boldsymbol{x} \right) \quad \textbf{(S7)}$$

subject to

$$\mathbf{S}(\rho(\mathrm{F}(\xi(\boldsymbol{x}))), \kappa(\mathrm{F}(\xi(\boldsymbol{x}))), \omega_j)\ \mathbf{p} = \mathbf{f}(\omega_j) \quad \textbf{(S8)}$$

$$0 < \xi(\boldsymbol{x}) < 1 \quad \textbf{(S9)}$$

Here ω_j denotes the frequencies targeted in the optimization problem, p_target the desired pressure field as a function of frequency and $\boldsymbol{\Omega}_\mathrm{t}$ the region of space in which p is sought matched to p_target. By choosing different $p_\mathrm{target}(\omega_j)$ expressions it is possible to design different devices with a variety of emission patterns. For both the ARE and the lambda-splitter $p_\mathrm{target}(\omega_j)$ is chosen as a spatially confined propagating plane wave with a frequency dependent propagation direction,

$$p_\mathrm{target}(\omega_j) = A_j\ \mathrm{e}^{-\mathrm{i}\,\omega_j\,(\boldsymbol{x}\cdot\boldsymbol{d}_j)}\ \mathrm{e}^{-\frac{\left|(\boldsymbol{x}-\boldsymbol{x}_\mathrm{w,j}) - \left((\boldsymbol{x}-\boldsymbol{x}_\mathrm{w,j})\cdot\boldsymbol{d}_j\right)\cdot\boldsymbol{d}_j\right|^2}{\delta_\mathrm{w,j}{}^2}}\ \mathrm{H}\left((\boldsymbol{x}-\boldsymbol{x}_\mathrm{w,j})\cdot\boldsymbol{d}_j\right). \quad \textbf{(S10)}$$

Here $\boldsymbol{d}_j$ denotes the propagation direction, $\boldsymbol{x}_\mathrm{w,j}$ the center of the Gaussian envelope localizing p_target in space, $\delta_\mathrm{w,j}$ the width of the envelope, and H a Heaviside function used to impose that p_target is zero in the half plane opposite the propagation direction.

The optimization problem **(S7-S9)** is solved with the globally convergent method of moving asymptotes *(46)*, using a maximum of three inner iterations per outer iteration.

Emission efficiency. The proposed design problem formulation enables the design of devices with high emission efficiency, because the formulation considers the minimization of the difference between the field emitted from the device under design for

a given material configuration and a target field freely chosen by the designer. This means that the designer is able to target implicitly a specific emission efficiency by choosing the total emitted power through the choice of p_{target} in **(S10)**. In the case of the ARE the choice of the amplitude, A, of p_{target} as well as the width of the Gaussian envelope, δ_w, controls the targeted emitted power. By selecting an appropriate value of A the solution of **(S7-S9)** implicitly leads to an emission efficiency above that of a point source emitting into free space. The physical explanation is that the device being designed not only controls the emission pattern of the emitted field, but also efficiently acts as an impedance matching mechanism between the source and the surrounding medium.

To demonstrate the high emission efficiency of the ARE (Fig. 2A) we use a Helmholtz-equation-based model of the physics, which is as close to the experimental setup for the fabricated sample (Fig. 2B) as possible. To this end, we construct a 3D model of the geometry using COMSOL Multiphysics. Here the 2D ARE is modelled as a set of hard wall boundaries, which are extruded in the out-of-plane direction to 6 mm height. The extruded ARE is then sandwiched between two hard boundaries in the out-of-plane direction. The in-plane boundaries of the model domain are truncated using perfectly matched layers to avoid reflections. In this model the ARE is excited through a hard-walled tube of 1.5 mm radius and length 6 mm, connected to the ARE at its center, which in turn is excited at the free end using a simple acceleration boundary condition. As a reference, we construct an identical model, except with the ARE removed. Both models are solved for a set of frequencies between 7.6 kHz and 12.8 kHz and the total emitted

far-field power is computed at each frequency. The result of these computations are presented in fig. S2. From the figure, it is seen that the total emitted power for the model with the ARE is higher than for the reference model over the entire frequency band from 7.6 kHz through 12.8 kHz. Hence, relative to the empty reference setup, above unity emission efficiency is achieved.

Experimental Setup and Procedure. The sound emission from the ARE and the lambda-splitter is measured experimentally using the setup described in *(44)*. In brief, the setup consists of a flat test chamber with a sound hard floor containing a cutout for test specimens at its center. The chamber is designed to exhibit near-anechoic termination at its sidewalls for frequencies above 5 kHz. It is sealed by a sound-hard, movable, ceiling with a flush-mounted 1/8th inch microphone at its center. The chamber supports two-dimensional sound propagation up to a frequency of $\cong$ 28.5 kHz, above which the sound field becomes fully three-dimensional. The near-anechoic termination is achieved using a layer of absorbing foam. The mono-polar sound source at the center of the optimized device is approximated by a hole (1.5 mm radius) through the bottom of the test specimens (see Fig. 2b and Fig. 3b) into which sound is funneled from a loudspeaker through a long waveguide. The loudspeaker is connected to a preamplifier driven by pseudorandom noise bandlimited to 6.8-13.2 kHz for the ARE measurements and to 6.0-12.4 kHz for the lambda-splitter measurements, respectively.

3D printing is used for production of the test specimens, based on the blueprints shown in Fig. 2a and Fig. 3a, respectively. The blueprints are extruded to a height of 6 mm on top

of a baseplate of 10 mm height to fit the test chamber dimensions. The lambda-splitter is produced in acrylonitrile butadiene styrene plastic (0.33 mm accuracy), and the rainbow is produced in photopolymer resin by Formlabs (0.2 mm accuracy). An image of the experimental setup with the ARE installed in the chamber is provided in fig. S3.

The sound emitted from the specimen is measured using the flush-mounted microphone by scanning it through a -90° to +90° angular interval using 2.5° +/- 0.25° increments over a half-circle with a fixed radius of 0.22 m +/- 0.002 m, centered at the hole in the specimen. Each pressure measurement is reported utilizing 250 averages.

The sound power emitted by the source varies with frequency due to the resonances of the long waveguide, causing significant frequency-dependent variations in its termination impedance. This variation has been corrected for in the experimental data presented in Fig. 2d and Fig. 3d as follows: The sound pressure magnitude measured for each frequency, is scaled by the root-mean-square pressure as,

$$|p_{\text{corrected}}(\theta, f)| = |p_{\text{measured}}(\theta, \text{f})| \Big/ \sqrt{\sum_{i=1}^{n_\theta} (|p_{\text{measured}}(\theta_i, f)|)^2 / n_\theta}\,, \qquad \textbf{(S12)}$$

where n_θ denotes the number of angular measurements. This undesirable frequency dependent sound-pressure variation can be removed, either by installing the loudspeaker directly at the center of the test specimen, or by correcting the input power to the system according to the termination impedance of the waveguide.

Rainbow illustration. The rainbow visualization seen in Fig. 1 is created by mapping the experimentally measured (termination impedance corrected) sound power over the

frequency spectrum [7600 kHz, 12800 kHz] to the visible spectrum of light [390 nm, 700 nm].

The CIE 1931 XYZ standard color space converted to the RGB color space using MATLAB v2017a's xyz2rgb function (assuming d65-illumination), is used to compute the angularly dependent color distribution as

$$R(\theta_j) = \sum_i r(f_i)\, P(f_i, \theta_j)\Delta f \quad \textbf{(S13)}$$

$$G(\theta_j) = \sum_i g(f_i)\, P(f_i, \theta_j)\Delta f \quad \textbf{(S14)}$$

$$B(\theta_j) = \sum_i b(f_i)\, P(f_i, \theta_j)\Delta f \quad \textbf{(S15)}$$

Here f_i denotes the measurement frequencies and θ_j denotes the measurement angles, P is the sound power, r, g and b are the RGB color-matching functions and $R(\theta_j)$, $G(\theta_j)$ and $B(\theta_j)$ are the resulting angularly dependent RGB-values.

The computation leads to negative RGB-values due to highly saturated colors (sound) at certain angles. As negative RGB-values cannot be displayed they are removed utilizing desaturation by adding a constant white light at all angles corresponding to the most negative RGB-value. The resulting RGB-spectrum is finally normalized to the interval [0,1].

The superimposed sinusoidal waves show the targeted wavelength as a function of angle. The amplitude of the waves is scaled according to the total measured sound power at each angle and decays according to the inverse square law.

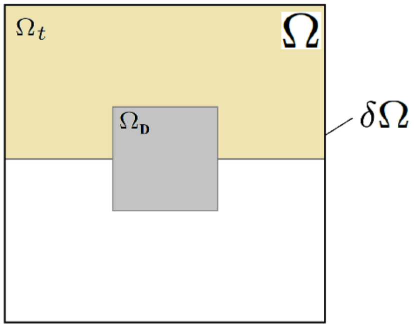

figure S1: Simple sketch of model domain, design domain and target domain.

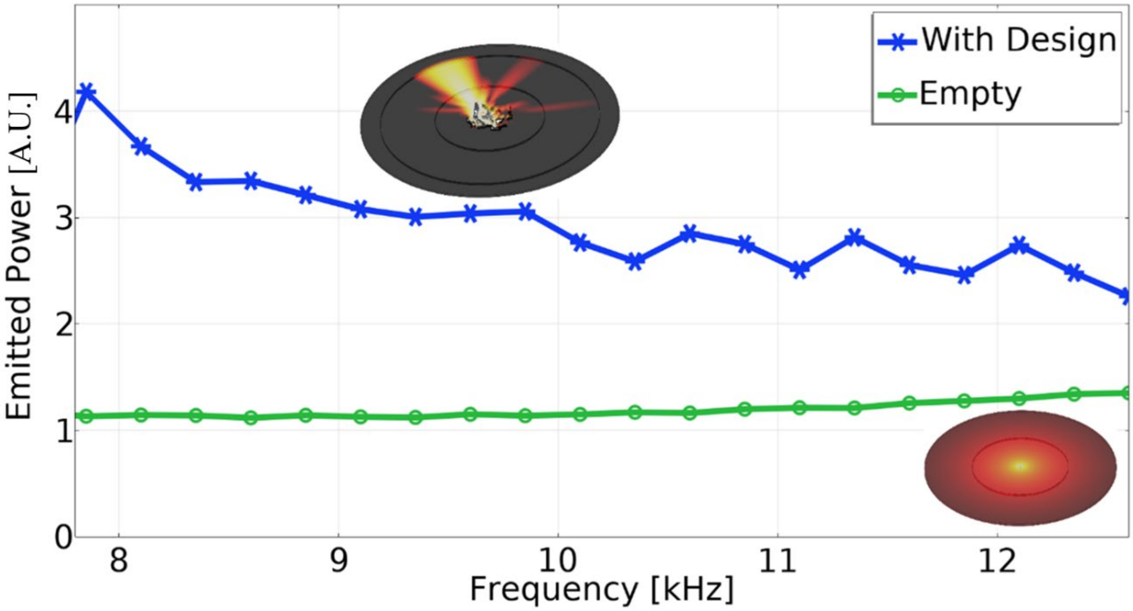

figure S2. Numerically evaluated emission efficiency of ARE (blue) and a reference without a design (red).

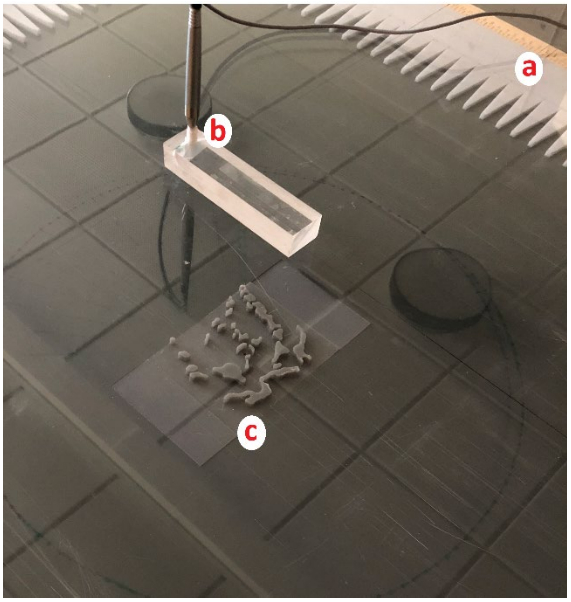

figure S3. Picture of the flat approximately 2D near-anechoic chamber. (a) Absorbing foam walls with wedges for added sound absorption. (b) Flush mounted microphone. (c) Test specimen.

movie S1. Animation of the spatial near-field sound pressure as a function of frequency for the ARE. The ARE is shown in white with the sound-pressure level is shown using a heat colormap on a 20 dB scale. The animation shows how the near-field changes with frequency, clearly revealing the controlled spatio-spectral separation of the emitted sound.

movie S2. Animation of the spatial near-field sound pressure as a function of frequency for the Lambda-Splitter. The lambda-splitter is shown in white with the sound-pressure level is shown using a heat colormap on a 20 dB scale. The animation

shows how the near-field changes with frequency, clearly revealing the controlled spatio-spectral separation of the emitted sound.

movie S3. Animation of a rotating ARE driven by broad-band white-noise and the audio heard by an observer. The ARE (white) is shown rotating relative to an observer (red dot) with the far-field sound pressure mapped to the optical rainbow and the sound heard by the observer under white-noise excitation of the ARE played as audio.

movie S4. Map of the far-field sound-pressure for the ARE driven by broad-band white-noise with animated observer angle and associated audio. The sound-pressure map from fig. 2C with an superimposed observer angle swept across the map with the associated audio heard by the observer.

REFERENCES AND NOTES

1. I. Newton, *Opticks, or a Treatise of the Reflections, Refractions, Inflections and Colours of Light* (Royal Society, 1703).

2. J. Zhu, Y. Chen, X. Zhu, F. J. Garcia-Vidal, X. Yin, W. Zhang, X. Zhang, Acoustic rainbow trapping. *Sci. Rep.* **3**, 1728 (2013).

3. V. Smolyaninova, I. Smolyaninov, A. Kildishev, V. Shalaev, Experimental observation of the trapped rainbow. *Appl. Phys. Lett.* **96**, 211121 (2010).

4. N. Jiménez, V. Romero-Garcia, V. Pagneux, J.-P. Groby, Rainbow-trapping absorbers: Broadband, perfect and asymmetric sound absorption by subwavelength panels for transmission problems. *Sci. Rep.* **7**, 13595 (2017).

5. A. Karlos, S. J. Elliott, Cochlea-inspired design of an acoustic rainbow sensor with a smoothly varying frequency response. *Sci. Rep.* **10**, 1–11 (2020).

6. M. L. Munjal, *Acoustics of Ducts and Mufflers* (Wiley and Sons, ed. 2, 2014).

7. R. D. White, K. Grosh, Microengineered hydromechanical cochlear model. *Proc. Natl. Acad. Sci. U.S.A.* **102**, 1296–1301 (2005).

8. R. Fleury, D. L. Sounas, C. F. Sieck, M. R. Haberman, A. Alù, Sound isolation and giant linear nonreciprocity in a compact acoustic circulator. *Science* **343**, 516–519 (2014).

9. Z. Liang, J. Li, Extreme acoustic metamaterial by coiling up space. *Phys. Rev. Lett.* **108**, 114301 (2012).

10. V. Romero-García, C. Lagarrigue, J.-P. Groby, O. Richoux, V. Tournat, Tunable acoustic waveguides in periodic arrays made of rigid square-rod scatterers: Theory and experimental realization. *J. Phys. D Appl. Phys.* **46**, 305108 (2013).

11. Y. Xie, C. Shen, W. Wang, J. Li, D. Suo, B. I. Popa, Y. Jing, S. A. Cummer, Acoustic holographic rendering with two-dimensional metamaterial-based passive phased array. *Sci. Rep.* **6**, 35437 (2016).

12. A. Håkansson, J. Sánchez-Dehesa, F. Cervera, Experimental realization of sonic demultiplexing devices based on inverse designed scattering acoustic elements. *Appl. Phys. Lett.* **88**, 163506 (2006).

13. A. Bozhko, J. Sánchez-Dehesa, F. Cervera, A. Krokhin, Redirection and splitting of sound waves by a periodic chain of thin perforated cylindrical shells. *Phys. Rev. Appl.* **7**, 064034 (2017).

14. H. Esfahlani, S. Karkar, H. Lissek, J. R. Mosig, Acoustic dispersive prism. *Sci. Rep.* **6**, 18911 (2016).

15. C. J. Naify, C. N. Layman, T. P. Martin, M. Nicholas, D. C. Calvo, G. J. Orris, Experimental realization of a variable index transmission line metamaterial as an acoustic leaky-wave antenna. *Appl. Phys. Lett.* **102**, 203508 (2013).

16. G. J. Chaplain, J. M. D. Ponti, G. Aguzzi, A. Colombi, R. V. Craster, Topological rainbow trapping for elastic energy harvesting in graded Su-Schrieffer-Heeger systems. *Phys. Rev. Appl.* **14**, 054035 (2020).

17. Z. Tian, C. Shen, J. Li, E. Reit, H. Bachman, J. E. S. Socolar, S. A. Cummer, T. J. Huang, Dispersion tuning and route reconfiguration of acoustic waves in valley topological phononic crystals. *Nat. Commun.* **11**, 762 (2020).

18. W. Yost, G. Gourevitch, *Directional Hearing* (Springer-Verlag, 1987).

19. P. Hofman, J. V. Riswick, A. V. Opstal, Relearning sound localization with new ears. *Nat. Neurosci.* **1**, 417–421 (1998).

20. M. Linnenschmidt, L. Wiegrebe, Sonar beam dynamics in leaf-nosed bats. *Sci. Rep.* **6**, 29222 (2016).

21. B. K. Branstetter, P. W. Moore, J. J. Finneran, M. N. Tormey, H. Aihara, Directional properties of bottlenose dolphin (*Tursiops truncatus*) clicks, burst-pulse, and whistle sounds. *J. Acoust. Soc. Am.* **131**, 1613–1621 (2012).

22. M. O. Lammers, W. W. L. Au, Directionality in the whistles of Hawaiian spinner dolphins (*Stenella longirostris*): A signal feature to cue direction of movement? *Mar. Mamm. Sci.* **19**, 249–264 (2003).

23. P. J. O. Miller, Mixed-directionality of killer whale stereotyped calls: A direction of movement cue? *Behav. Ecol. Sociobiol.* **52**, 262 (2002).

24. M. Dantzker, G. Deane, J. Bradbury, Directional acoustic radiation in the strut display of male sage grouse Centrocercus urophasianus. *J. Exp. Biol.* **202**, (Pt 21) 2893–2909 (1999).

25. M. N. Coleman, C. F. Ross, Primate auditory diversity and its influence on hearing performance. *Anat. Rec. A Discov. Mol. Cell. Evol. Biol.* **281**, 1123–1137 (2004).

26. E. Dong, Y. Zhang, Z. Song, T. Zhang, C. Cai, N. X. Fang, Physical modeling and validation of porpoises' directional emission via hybrid metamaterials. *Natl. Sci. Rev.* **6**, 921–928 (2019).

27. E. Dong, Y. Zhou, Y. Zhang, H. Chen, Bioinspired conformal transformation acoustics. *Phys. Rev.Appl.* **13**, 024002 (2020).

28. M. P. Bendsøe, O. Sigmund, *Topology Optimization* (Springer, 2003).

29. N. Aage, E. Andreassen, B. S. Lazarov, O. Sigmund, Giga-voxel computational morphogenesis for structural design. *Nature* **550**, 84–86 (2017).

30. J. Alexandersen, O. Sigmund, N. Aage, Large scale three-dimensional topology optimisation of heat sinks cooled by natural convection. *Int. J. Heat Mass Transf.* **100**, 876–891 (2016).

31. J. Jensen, O. Sigmund, Topology optimization for nano-photonics. *Laser Photon. Rev.* **5**, 308–321 (2011).

32. M. B. Dühring, J. S. Jensen, O. Sigmund, Acoustic design by topology optimization. *J. Sound Vib.* **317**, 557–575 (2008).

33. E. Wadbro, M. Berggren, Topology optimization of an acoustic horn. *Comput. Methods Appl. Mech. Eng.* **196**, 420–436 (2006).

34. W. Li, F. Meng, Y. Chen, Y. F. Li, X. Huang, Topology optimization of photonic and phononic crystals and metamaterials: A review. *Adv. Theory Simul.* **2**, 1900017 (2019).

35. H. W. Dong, S. D. Zhao, P. Wei, L. Cheng, Y. S. Wang, C. Zhang, Systematic design and realization of double-negative acoustic metamaterials by topology optimization. *Acta Mater.* **172**, 102–120 (2019).

36. H. W. Dong, S. D. Zhao, Y. S. Wang, L. Cheng, C. Zhang, Robust 2D/3D multi-polar acoustic metamaterials with broadband double negativity. *J. Mech. Phys. Solids* **137**, 103889 (2020).

37. J. Rong, W. Ye, Topology optimization design scheme for broadband non-resonant hyperbolic elastic metamaterials. *Comput. Methods Appl. Mech. Eng.* **344**, 819–836 (2019).

38. R. E. Christiansen, F. Wang, O. Sigmund, Topological insulators by topology optimization. *Phys. Rev. Lett.* **122**, 234502 (2019).

39. Y. Chen, F. Meng, X. Huang, Creating acoustic topological insulators through topology optimization. *Mech. Syst. Signal Process.* **146**, 107054 (2021).

40. C. B. Dilgen, S. B. Dilgen, N. Aage, J. S. Jensen, Topology optimization of acoustic mechanical interaction problems: a comparative review. *Struct. Multidiscipl. Optim.* **60**, 779–801 (2019).

41. C. B. Dilgen, N. Aage, Topology optimization of transient vibroacoustic problems for broadband filter design using cut elements. *FEAD* **234**, 104123 (2024).

42. V. Cool, O. Sigmund, N. Aage, F. Naets, E. Deckers, Vibroacoustic topology optimization for sound transmission minimization through sandwich structures. *J. Sound Vib.* **568**, 117959 (2024).

43. F. Jacobsen, P. M. Juhl, *Fundamentals of General Linear Acoustics* (Wiley, 2013).

44. R. E. Christiansen, E. Fernandez-Grande, Design of passive directional acoustic devices using topology optimization—From method to experimental validation. *J. Acoust. Soc. Am.* **140**, 3862–3873 (2016).

45. D. A. Tortorelli, P. Michaleris, Design sensitivity analysis: Overview and review. *Inverse Probl. Eng* **1**, 71–105 (1994).

46. K. Svanberg, A class of globally convergent optimization methods based on conservative convex separable approximations. *SIAM J. Optim.* **12**, 555–573 (2002).

47. R. E. Christiansen, O. Sigmund, E. Fernandez-Grande, Experimental validation of a topology optimized acoustic cavity. *J. Acoust. Soc. Am.* **138**, 3470–3474 (2015).

48. H. Takemoto, P. Mokhtari, H. Kato, R. Nishimura, K. Iida, Mechanism for generating peaks and notches of head-related transfer functions in the median plane. *J. Acoust. Soc. Am.* **132**, 3832–3841 (2012).

49. S. Spagnol, F. Avanzini, "Frequency estimation of the first pinna notch in head-related transfer functions with a linear anthropometric model," *Proceedings of the International Conference on Digital Audio Effects* (2015), Department of Music and Department of Electronics and Telecommunications, Norwegian University of Science and Technology, pp. 231–236.

50. COMSOL AB, COMSOL Multiphysics v. 6.1; www.comsol.com.

51. G. Seongyeol, W. Semyung, K. Junghwan, K. Kunmo, H. Jaeyub, Topology optimization of bounded acoustic problems using the hybrid finite element-wave based method. *Comput. Methods Appl. Mech. Eng.* **313**, 834 (2017).

52. B. Pluymers, "Wave based modelling methods for steady-state vibro-acoustics," thesis, KULeuven (2006).
